# Risk Factors for Urinary Incontinence in Pregnancy: A Case Control Study

**DOI:** 10.1055/s-0040-1718951

**Published:** 2020-12-21

**Authors:** Fernanda Borsatto Caruso, Lucas Schreiner, Alexandra Damasio Todescatto, Isabel Crivelatti, Julia Monteiro de Oliveira

**Affiliations:** 1 Pontifícia Universidade Católica do Rio Grande do Sul, Porto Alegre, RS, Brazil

**Keywords:** urinary incontinence, pregnancy, pelvic floor, tobacco use, incontinência urinária, gestação, assoalho pélvico, tabagismo

## Abstract

**Objective** Urinay incontinence (UI) is a major public health problem that can harm women in any period of life, including during the gestational period. Urinary incontinence during pregnancy has been studied because this condition can reduce the quality of life and interfere in several aspects of the maternal-fetal binomial. The aim of this study was to determine the prevalence of UI in nullipara pregnant women and to identify risk factors associated with UI in this population.

**Methods** This is a case-control study in which we invited nullipara women between 12 and 20 weeks of pregnancy to participate in the research. They were asked to answer a specific questionnaire, write a 3-day bladder diary, and undergo a urogynecological evaluation including pelvic organ prolapse quantification (POP-Q), empty stress supine test (ESST), and pelvic floor muscle assessment.

**Results** A total of 70 out of 73 patients accepted to participate in the study, and the prevalence of UI in this population was 18.3%. Tobacco use was identified as an independent risk factor for UI in pregnant women (odds ratio 8.0). All other factors analyzed were not significantly associated to UI in pregnancy.

**Conclusion** Urinary incontinence can be a major problem in pregnancy. We identified the use of tobacco as a risk factor for developing UI in pregnancy, which provides an extra reason to encourage patients to quit smoking.

## Resumo

**Objetivo** A incontinência urinária (IU) é um importante problema de saúde pública que pode trazer prejuízos às mulheres em qualquer período da vida, inclusive durante o período gestacional. A IU durante a gravidez tem sido estudada por ser capaz de reduzir a qualidade de vida e interferir em vários aspectos do binômio materno-fetal. O objetivo deste estudo foi determinar a prevalência de IU em gestantes nulíparas e identificar fatores de risco associados a essa população.

**Métodos** Este é um estudo de caso-controle em que foram convidadas mulheres nulíparas entre 12 e 20 semanas de gravidez para participar do projeto. Elas foram submetidas a um questionário específico, diário miccional de 3 dias e avaliação uroginecológica, incluindo quantificação de prolapso de órgãos pélvicos (POP-Q), teste de esforço com volume residual e avaliação da musculatura do assoalho pélvico.

**Resultados** Um total de 70 das 73 pacientes aceitaram participar do estudo, e a prevalência de incontinência urinária nessa população foi de 18,3%. O uso de tabaco foi identificado como fator de risco independente para a IU em gestantes (OR 8,0). Todos os outros fatores analisados não foram significativamente associados à perda urinária nessa população.

**Conclusão** A incontinência urinária pode trazer prejuízos para pacientes durante o período da gestação. O tabagismo foi identificado como fator de risco para o desenvolvimento de IU em gestantes, o que denota mais um motivo para encorajar as pacientes a abandonarem o hábito.

## Introduction

Pregnancy is one of the main risk factors for the development of urinary incontinence (UI) in young women. Physiological changes during pregnancy, such as increasing pressure of the growing uterus and fetal weight on the pelvic floor muscle (PFM) throughout pregnancy, together with pregnancy-related hormonal changes such as increased progesterone, decreased relaxin, and decreased collagen levels, may lead to reduced strength and supportive and sphincteric function of the PFM. Pregnancy may associate with the reduction of the PFM strength, which can lead to stress urinary incontinence (SUI). 1

The prevalence of UI among pregnant women has been found to be from 18 to 75%; it increases with gestational age and is typically worst in the third trimester followed by second and first trimesters, respectively. 2 3 4

Studies in pregnant women with SUI have found significantly decreased PFM strength in incontinent pregnant women as compared with continent pregnant women. 5

Although UI is non-life threatening, the effect on the women's quality of life can be substantial. Women with moderate-to-severe UI may suffer from emotional disorders, social embarrassment, loss of self-esteem, and have sexual relationship difficulties, since many present loss of urine during the sexual act. 6 7

The symptoms could worse after the gestational period and are mainly related with delivery. A systematic review and metanalysis of women with UI, with a follow-up period longer than 1 year after delivery, showed that vaginal delivery was associated with a 2-fold risk of developing UI compared with cesarean section (CS) and a 3-fold risk compared with elective CS. 8

This study has the objective to evaluate the prevalence and the main risk factors for developing UI in nulliparas women. This period is commonly very important and expected for these patients, and the loss of urine could be distressing, reducing quality of life and interfering in several aspects of the maternal–fetal binomial.

## Methods

This is a case control study performed at Hospital São Lucas from the *Pontifícia Universidade Católica do Rio Grande do Sul* (PUCRS) between January and June 2017. We prospectively invited all pregnant patients who started antenatal follow-up and included those between 12 and 20 weeks of pregnancy, focusing on factors strictly related to their current pregnancy. We excluded those patients in the first trimester of pregnancy to avoid cases of miscarriage in the current pregnancy and those in the third trimester, because this is when the women have greater weight gain and physiological changes, which are confounders to urinary symptoms. Only single pregnancies were included. The exclusion criteria were previous delivery, neurological disease, previous surgery for pelvic floor dysfunction, UI prior to the current pregnancy, chronic diabetes, and previous pelvic radiotherapy. Institutional Review Board approval was obtained prior to the beginning of the study.

All subjects completed a specific questionnaire (International Consultation on Incontinence Questionnaire-Urinary Incontinence Short Form [ICIQ-UI SF]) and underwent physical exam including urogynecological evaluation (Pelvic organ prolapse - quantification, empty stress supine test, and PFM assessment).

The patients who were diagnosed with UI (ICIQ-UI SF score ≥ 3) were referred to physiotherapy treatment.

All data were anonymous and analyzed using the SPSS 17.0 software (SPSS Inc., Chicago, IL, USA). Univariate logistic regression analysis was performed with respect to UI and possible predictive factors. Descriptive analysis was performed using frequencies, means, and standard deviations. The Kolmogorov-Smirnov test was used to evaluate if the data had a normal distribution. For comparison between groups, we used the following tests: Chi-squared or Fisher exact test for categorical variables, and Student *t* -test for independent samples to verify difference between averages. Associations were considered statistically significant if *p* -value < 0.05.

Smoking was the only risk factor with statistical significance for UI in pregnancy, 57.1% of UI in smokers versus 14.3% in non-smokers OR 8.0. ( Table 3 ). Among the incontinent patients, 76.9% complained of SUI, and 53.8% complained of urgency. When we calculated the patients' body mass index (BMI), we found a higher mean BMI in incontinent patients (33.3 kg/m ^2^ incontinent vs 29.5 kg/m ^2^ in the continents), but the difference was not statistically significant in the sample. We invited 73 women to participate in the study according to our inclusion and exclusion criteria, 3 patients did not accept/ did not have time (estimated in 60 minutes plus the time required to write a bladder diary) available to complete the protocol. Our sample was 70 pregnant women between 12 and 20 weeks of pregnancy. The prevalence of incontinence in our study was 18.3%. In our group of 70 patients, 74.3% were white, 4.3% had diabetes, 10.1% had high blood pressure, and 10% were tobacco users. Regarding previous procedures, 18.6% of the patients revealed previous miscarriage, and 15.7% underwent previous surgery (exception for exclusion criteria) before pregnancy ( Table 1 ). During evaluation of the PFM strength, all patients recognized the PFM, 43.3% presented weak Kegel, 47.3% presented normal Kegel, and 9% strong Kegel. There was no difference in the PFM strength between continent and incontinent pregnant women. There was no difference in mean age and year of study between groups (25.9 years in incontinent group × 24.9 years in control). Regarding the number of years of study, the average in the incontinent group was 10.3 years, and 10.7 years in the continent group ( Table 2 ). The frequency of micturition per day in the incontinent group was 9.8 times, whereas among the continent group it was 8.4 times. Meanwhile, mean nocturia was 2.8 in the incontinent group and 2.1 in the control group. The length of the genital hiatus had a mean of 2.4 cm in both groups. The mean perineal length in the incontinent group was 3.8 cm, and 3.4 cm in the control group. In the incontinent group, the mean total vaginal length was 9.7 cm, while in the continent women it was 10.6 cm. There was no difference between bladder diary parameters and POP-Q measures between continent and incontinent pregnant women ( Table 2 ). Smoking was the only risk factor with statistical significance for UI in pregnancy, with 57.1% of UI in smokers versus 14.3% in non-smokers (odds ratio [OR] 8.0.) ( Table 3 ).

**Table 1:** Frequencies of demographic and clinical characteristics in our study group

Variable	Total ( *n* = 70) %
**White race**	74.3
**Diabetes**	4.3
**Arterial hypertension**	10.1
**Smoking**	10.0
**Previous abortion**	18.6
**Previous surgery to gestation**	15.7

**Table 2:** Urinary incontinence in numeric variables according to univariate analysis (n = 70)

Variable	Urinary incontinence		*p* *
	yes	No	
	(n = 13)	(n = 57)	
	x ± sd	x ± sd	
**Age**	25.9 ± 5.5	24.9 ± 6.6	0.624
**Time with partner (in months)**	56.3 ± 34.4	43.5 ± 37.9	0.271
**Years of study**	10.3 ± 3.1	10.7 ± 2.0	0.622
**Number of previous pregnancies**	1.2 ± 0.4	1.2 ± 0.5	0.986
**Number of previous abortions**	0.5 ± 0.8	1.1 ± 0.4	0.075
**Daytime urinary frequency**	9.8 ± 5.4	8.4 ± 3.4	0.377
**Nocturia**	2.8 ± 2.1	2.1 ± 1.9	0.233
**Genital hiatus (cm)**	2.4 ± 0.5	2.4 ± 0.6	0.906
**Perineal body (cm)**	3.8 ± 0.6	3.4 ± 0.8	0.212
**Total vaginal length (cm)**	9.7 1.7	10.6 ± 1.5	0.111
**BMI (Kg/m2)**	33.3 ± 8.8	29.5 ± 8.4	0.222

Abbreviations: BMI, body mass index; cm, centimeters; n, number; SD, standard deviation.

* *P* -value calculated by Student *t* -test for independent samples.

x ± SD - mean ± standard deviation.

**Table 3:** Risk of urinary incontinence during pregnancy in all patients according to univariate analysis in categorical variables ( *n* = 70)

Variable	Urinary incontinence %	OR	CI 95%	*p* -value
Tobacco Use	18.3 **			
Yes	57.1	8.0	(1.52–41.86)	0.019 ***
No	14.3			
Diabetes	18.3			
Yes	33.3	2.2	(0.18–26.88)	0.512 ***
No	18.2			
HAS	18.3			
Yes	14.3	0.6	(0.07–6.32)	0.745 ***
No	19.4			
Abortion	18.3			
Yes	23.1	1.4	(0.32–6.06)	0.643 *
No	17.5			
Previous surgery	18.3			
Yes	9.1	0.4	(0.04–3.36)	0.378 *
No	20.3			

Abbreviations: CI, confidence interval; OR, odds ratio.

* *PPP* -value calculated by the Chi-squared test of Pearson.

** Global risk of urinary incontinence (70 patients).

*** *P* -value calculated by Fisher exact test.

## Results

We invited 73 women to participate in the period of study according to our inclusion and exclusion criteria, 3 patients do not accept - do not have time (estimated in 60 minutes more bladder diary) available to complete the protocol. Our sample was 70 pregnant women between 12 and 20 weeks of pregnancy. The prevalence of incontinence in our study was 18.3% (13 patients). In our group of 70 patients, 74.3% were white, 4.3% had gestational diabetes, 10.1% high blood pressure and 10% were tobacco users. In respect of previous procedures, 18.6% of the patients revealed previous miscarriage and 15.7% underwent any surgery (exception for exclusion criteria) before pregnancy ( Table 1 ).

During evaluation of pelvic floor muscle strength, all patients recognize pelvic floor muscle, 43.3% presented Kegel weak, 47.3% Kegel normal and 9% Kegel strong. There was no difference between pelvic floor muscle strength between continent and incontinent pregnant women. There was no difference in mean age and year of study among groups (25.9 years in incontinent group × 24.9 years in control). In years of study, the average in incontinent group was 10.3 years and 10.7 years in continent group ( Table 2 ).

The frequency of micturition per day among incontinent group was 9.8 times whereas among continent was 8.4 times. Meanwhile, mean nocturia was 2.8 for incontinent and 2.1 for control. The length of the genital hiatus had a mean of 2.4 cm in both groups. The mean perineal length between incontinent group was 3.8 cm and between control 3.4. Among the incontinent group, the mean total vaginal length was 9.7 cm, while among non-incontinent women it was 10.6 cm. There was no difference between bladder diary parameters and POP-Q measures between continent and incontinent pregnant women ( Table 2 ). Smoking was the only risk factor with statistical significance for UI in pregnancy, 57.1% of UI in smokers versus 14.3% in non-smokers OR 8.0 ( Table 3 ). Among the incontinent patients, 76.9% complained of SUI and 53.8% of urgency when we calculated the patients' body mass index (BMI), we found a higher mean BMI in incontinent patients (33.3 kg/m ^2^ incontinent vs 29.5 kg/m ^2^ in the continents), but the difference was not statistically significant in the sample.

Smoking was the only risk factor with statistical significance for UI in pregnancy, 57.1% of UI in smokers versus 14.3% in non-smokers OR 8.0. ( Table 3 ).Among the incontinent patients, 76.9% complained of SUI and 53.8% of urgency When we calculated the patients' body mass index (BMI), we found a higher mean BMI in incontinent patients (33.3 kg/m ^2^ incontinent vs 29.5 kg/m ^2^ in the continents), but the difference was not statistically significant in the sample.

## Discussion

The prevalence of UI depends on the population, habits, economic issues, and, in some studies, it could reach 75% of pregnant women. 2 The majority of studies are focused on Europe and North America, and only a few studies have shown the numbers in South America. In our sample, we identified an 18.3% of UI in nulliparous women. The inclusion of patients in the second trimester aimed to correlate other possible risk factors than those related to increased intra-abdominal pressure observed in the third trimester of pregnancy

The parity is an important factor for the development of UI, mainly for SUI. In a recent meta-analysis, Zhou et al. 9 found that the risk of UI is increased in women with two or more deliveries in comparison with nulliparous woman.

The occurrence of UI changes during different periods of pregnancy. In a questionnaire-based pilot study, Beksac et al. 10 demonstrated that the prevalence of any UI in nulliparous pregnant women was 4.9%, 9.8%, and 26.2% at 11–14, ∼ 24, and ∼ 37 gestational weeks, respectively. Stress urinary incontinence (3.3%, 6.6%, and 16.4% at 11–14, ∼ 24 and ∼ 37 gestational weeks, respectively) was found to be the main type of UI, as reported previously. 11 12 Regarding age at pregnancy, we have some discussions in the literature. Zhu et al. 13 reported SUI during pregnancy being associated with advanced maternal age. The risk of SUI incidence was increased with maternal age (OR = 1.041; 95% CI 1.027–1.055). This finding was supported by Hvidman et al. 14 who found out that pregnant women aged 30 years and older to be at significantly greater risk for SUI than younger women.

Contrary to the idea that younger women could have an additional protection in PFMs and ligaments, some works have shown that a pelvic floor injury sustained in young females may be more significant, and young age may not be protective. In a study that used ultrasound to investigate the extent of levator ani damage in low-risk primiparous women based on age found an inverse relationship between age and severity of levator ani damage. 15

In our study, the only factor we could associate with risk to develop SUI was smoking. We found 53.1% of UI in the tobacco group versus 14.7% of UI in pregnant who did not smoke (OR 8.0, CI [1.52–41.86], *p* = 0.019), with statistical significance.

The carbon monoxide in cigarettes impairs oxygen transported to bodily tissues and results in muscle atrophy. The PFM is also affected. Smoking can cause coughing, chronic, and frequent coughing, thus increasing bladder pressure and exerting significant pressure on the PFM, which may lead to damaging the innervation to the PFM and aggravated SUI. Not only carbon monoxide but also nicotine has a stimulating effect on the detrusor muscle. 16

The anatomy of the pelvic floor and physiology of the lower urinary tract play an important role in the background of the knowledge of the continence mechanism. Additionally, when we measured the genital hiatus, the perineal body and the total vaginal length, we did not see any difference between the groups.

Women with diabetes mellitus (DM) were at greater risk of incontinence than women without DM. In addition, the risk and severity of incontinence increased with the duration of the disease, with greater risk for developing symptoms in women with DM for 5 or more years. 17 In comparison with type 2 diabetes, gestational diabetes mellitus (GDM) was not correlated as a risk factor to develop UI. Some studies have shown a small increase of risk in women with GDM, but the mechanism to this development remains unclear. 18 Most likely, this association is correlated with increased body mass index (BMI), obesity, and macrosomia of infants.

Obesity is a major risk factor for SUI in women. Obesity chronically strains and creates tension on the pelvic floor due to increased intra-abdominal pressure and may impair blood flow and nerve innervation to the bladder and urethra. Furthermore, obesity may increase pressure on the bladder, thereby affecting the neuromuscular function of the genital tract and contributing to pelvic floor and urethral dysfunction. 19 20 21

In another study, Hvidman et al. 14 demonstrated that pregnant women with prepregnancy BMI of more than 30 kg/m2 were associated with a high rate of SUI. Liang et al. 3 have shown women with a prepregnancy BMI of more than 30 kg/m2 to be at increased risk for developing SUI during pregnancy.

When analyzing the data in our study, the difference between the BMI of the groups was not shown to be statistically significant, but there was a trend toward significance, with a higher mean BMI in the patients presenting with incontinence. Obesity is a worldwide epidemic sickness. It is a risk factor for incontinence in patients of any age group, and this trend seems to be maintained during pregnancy and may even worsen due to the increase in intra-abdominal pressure caused by the gravid uterus.

In our study, the prevalence of UI was 18.3%, but, in contrast with most papers, we analyzed just nulliparous women in the second trimester of pregnancy. 22 23 24

Urinary incontinence has a high prevalence in the general world population, especially in women. 25 Although the causes of UI are not fully understood, in most cases they are composed by habits and modifiable variables, like smoking and obesity. It is unfair for them to continue to suffer when evidence has clearly shown that pelvic floor exercises (PFEs) performed antenatally can reduce the risk of developing UI and also improve the symptoms of UI by strengthening the PFMs. 6 7

## Conclusion

Our sample was not sufficient for subgroup analysis, which is a limitation of our study, but the strength of the present study is that we selected only nulliparas in the early second trimester of pregnancy reducing the bias related to previous deliveries, current miscarriage, and confounding factors in the late pregnancy as weight gain an physiological changes related to urinary symptoms. In the evaluation of the possible risk factors associated with the onset of UI in nulliparous women, smoking was reported more frequently among those patients with UI complaints than in those without UI complaints. Obesity, although not representing statistical significance in this study, showed a trend toward significance. Other variables, such as hypertension, GDM, and previous surgical procedures did not correlate with the development of UI in our sample. Urinary incontinence is an important health issue in the gestational period and needs to be considered during the prenatal care.
